# The *feoABC* Locus of *Yersinia pestis* Likely Has Two Promoters Causing Unique Iron Regulation

**DOI:** 10.3389/fcimb.2017.00331

**Published:** 2017-07-21

**Authors:** Lauren O'Connor, Jacqueline D. Fetherston, Robert D. Perry

**Affiliations:** Department of Microbiology, Immunology, and Molecular Genetics, University of Kentucky Lexington, KY, United States

**Keywords:** ferrous transport, plague, iron regulation, ferric uptake, promoter structure

## Abstract

The FeoABC ferrous transporter is a wide-spread bacterial system. While the *feoABC* locus is regulated by a number of factors in the bacteria studied, we have previously found that regulation of *feoABC* in *Yersinia pestis* appears to be unique. None of the non-iron responsive transcriptional regulators that control expression of *feoABC* in other bacteria do so in *Y. pestis*. Another unique factor is the iron and Fur regulation of the *Y. pestis feoABC* locus occurs during microaerobic but not aerobic growth. Here we show that this unique iron-regulation is not due to a unique aspect of the *Y. pestis* Fur protein but to DNA sequences that regulate transcription. We have used truncations, alterations, and deletions of the *feoA::lacZ* reporter to assess the mechanism behind the failure of iron to repress transcription under aerobic conditions. These studies plus EMSAs and DNA sequence analysis have led to our proposal that the *feoABC* locus has two promoters: an upstream P1 promoter whose expression is relatively iron-independent but repressed under microaerobic conditions and the known downstream Fur-regulated P2 promoter. In addition, we have identified two regions that bind *Y. pestis* protein(s), although we have not identified these protein(s) or their function. Finally we used iron uptake assays to demonstrate that both FeoABC and YfeABCD transport ferrous iron in an energy-dependent manner and also use ferric iron as a substrate for uptake.

## Introduction

Iron is an essential nutrient and plays an important role in the pathogenesis of many organisms. Mammals chelate inorganic iron and heme using ferritin and hemoglobin in intracellular environments while transferrin, lactoferrin, heme- and hemoglobin-binding proteins sequester it in extracellular environments. Successful pathogens are able to acquire iron from these sources using a number of mechanisms: (1) secreted siderophores (2) heme transporters (3) ferric and ferrous transporters, as well as (4) transferrin and lactoferrin binding proteins to acquire iron from the host (Skaar, 2010).

Iron acquisition is crucial for the pathogenesis of *Yersinia pestis*, the causative agent of bubonic, pneumonic, and septicemic plague (Perry and Fetherston, 1997). Although, *Y. pestis* has multiple uptake systems for acquiring iron, relatively few have been shown to play a role in virulence in mammals (Kirillina et al., 2004; Perry, 2004; Forman et al., 2010). The yersiniabactin (Ybt) siderophore system is one of the most important for obtaining ferric iron (Kirillina et al., 2006; Perry and Fetherston, 2011) and is critical for virulence during pneumonic and the early stages of bubonic plague (Bearden et al., 1997; Fetherston et al., 1999, 2010). Ybt is encoded on the chromosomal *pgm* locus, a 102 kb region that can be spontaneously deleted. Recently, Bobrov et al. showed that components of the Ybt system are also involved in zinc uptake (Bobrov et al., 2014). The functions of 4 loci within the *pgm* locus have been determined: (1) *ybt* which encodes genes for the biosynthesis, regulation, and transport of the Ybt siderophore, (2) *fet*-*flp*, which encodes a ferrous iron transport system, (3) *hms*, which encodes components for biofilm development and (4) *rip*, which is involved in intracellular survival in phagocytic cells (Jarrett et al., 2004; Kirillina et al., 2004; Pujol et al., 2009; Forman et al., 2010).

The Yfe, Yfu, Yiu, and Hmu systems are ABC transporters involved in iron or heme uptake (Perry et al., 1990; Darby et al., 2002; Kirillina et al., 2004; Forman et al., 2010). The Hmu system is functional *in vitro* and is essential for the use of heme, but it does not play a role in the progression of bubonic or pneumonic plague (Thompson et al., 1999; Forman et al., 2010). The Yfu and Yiu transporters are functional *in vitro*; however, they are not important for the disease progression of bubonic plague (Gong et al., 2001; Kirillina et al., 2006). The Yfe system is an ABC transporter that transports iron and manganese. YfeA is a periplasmic binding protein that can bind Fe, Mn, and Zn, however, only Fe and Mn can be transported into the cytoplasm through the inner membrane permeases, YfeC and YfeD, with the help of the ATPase, YfeB (Bearden and Perry, 1999; Perry et al., 2012). Bearden et al. demonstrated that in an Ybt^−^ background, a Δ*yfeAB* mutant had ~2.5- fold decrease in its ability to transport iron (Bearden and Perry, 1999). In 2012, Fetherston et al. showed that in a Ybt^−^ background, a Δ*yfeAB* Δ*feoB* mutant was completely defective in transporting ferrous iron (Fetherston et al., 2012). The Yfe and Feo systems appear to play somewhat of a redundant role *in vitro*, and play a role in the virulence of bubonic, but not pneumonic plague (Bearden and Perry, 1999; Fetherston et al., 2012).

The FeoABC system is comprised of three proteins. FeoB is an 85 kDa permease, which transports iron from the periplasm into the cytoplasm. FeoA is a small, 8.5 kDa protein that is predicted to interact with FeoB and stimulate GTPase activity. FeoC is a small, 9 kDa protein of unknown function. Due to its winged-helix motif, it has been predicted to play a role in regulation in *Escherichia coli* (Kammler et al., 1993; Lau et al., 2013). FeoA_*YP*_ and FeoB_*YP*_ have 77 and 74% identity to FeoA_*EC*_ and FeoB_*EC*_ respectively, whereas FeoC_*YP*_ only has 57% identity to FeoC_*EC*_ (Altschul et al., 1997). Guo et al. provided indirect evidence that FeoC is a negative regulator of the *feoABC* operon in *Y. pestis* (Gao et al., 2008). However, in our hands, a Δ*feoC* mutant did not affect transcription from the *feo* promoter under aerobic or microaerobic conditions (Fetherston et al., 2012). Due to our data, and the lack of homology between FeoC in *Y. pestis* and *E. coli*, we do not believe FeoC is a transcriptional repressor of the *feoABC* operon in *Y. pestis*. FeoC has been shown to contain an oxygen sensitive 4Fe-4S cluster and to bind to FeoB preventing its proteolytic degration by FtsH in *Klebsiella pneumoniae* (Hung et al., 2012; Hsueh et al., 2013; Kim et al., 2013). However, a similar role in *Y. pestis* is uncertain since the Feo system is functional in a Δ*feoC* mutant (Fetherston et al., 2012).

Iron is an essential element for the growth and pathogenesis of *Y. pestis*. However, excess iron in the presence of oxygen can lead to damaging reactive oxygen radicals. Therefore, iron transport into the cell is a very tightly regulated process. All of the tested ferrous and ferric iron uptake systems in *Y. pestis* are repressed by Fe-Fur under aerobic conditions, except for the FeoABC system. However, the *feoABC* locus is repressed by Fe under microaerobic conditions (Forman et al., 2010; Fetherston et al., 2012).

The *feoA* promoter in *Y. pestis* contains putative Fnr- and ArcA-binding sites (Gao et al., 2008). Previously, we have shown that Δ*fnr*, Δ*arcA*, and Δ*rstAB* mutations do not affect *feoB* transcription (Fetherston et al., 2012), even though Fnr positively regulates the *feo* operon of *E. coli* and *Shigella*, and ArcA and RstA are positive regulators of the operons in *Shigella* and *Salmonella*, respectively (Kammler et al., 1993; Boulette and Payne, 2007; Jeon et al., 2008).

In this study, we examined the transcriptional regulation of the *feoABC* operon. Our results suggest that a unique feature of the *feo* promoter region in *Y. pestis* prevents Fe-Fur repression aerobically but not microaerobically. We also demonstrate that the Yfe and Feo systems function in iron uptake under both reducing and non-reducing conditions.

## Materials and methods

### Bacterial strains and growth conditions

*Y. pestis* KIM6+ and derivatives of this strain (Table S1) were inoculated from −80°C glycerol stocks onto Congo Red plates. Plates were incubated at 30°C for ~48 h. Individual colonies were inoculated onto Tryptose Blood Agar Base (TBA) slants with the appropriate antibiotic, and incubated at 30°C overnight. *E. coli* strains were inoculated from −80°C glycerol stocks onto TBA slants with appropriate antibiotics and incubated at 30°C overnight. For transformants, *E. coli* cells were grown in Luria broth (LB) or on LB agar plates.

### Plasmid construction

#### 5′-truncations of the *Y. pestis* KIM6+ *feo* promoter region

Promoter truncations were constructed by PCR amplification using the primer pairs listed in Table S2. PCR products were digested with *Pme*I and *Asc*I and and cloned into the same sites of pWSK*feoA*::*lacZ* (Fetherston et al., 2012; Table S1). The resulting plasmids with the correct inserts were verified by sequencing and were electroporated into *Y. pestis* strains.

#### Construction of an *E. coli feoA* promoter reporter

**Figure 1:**
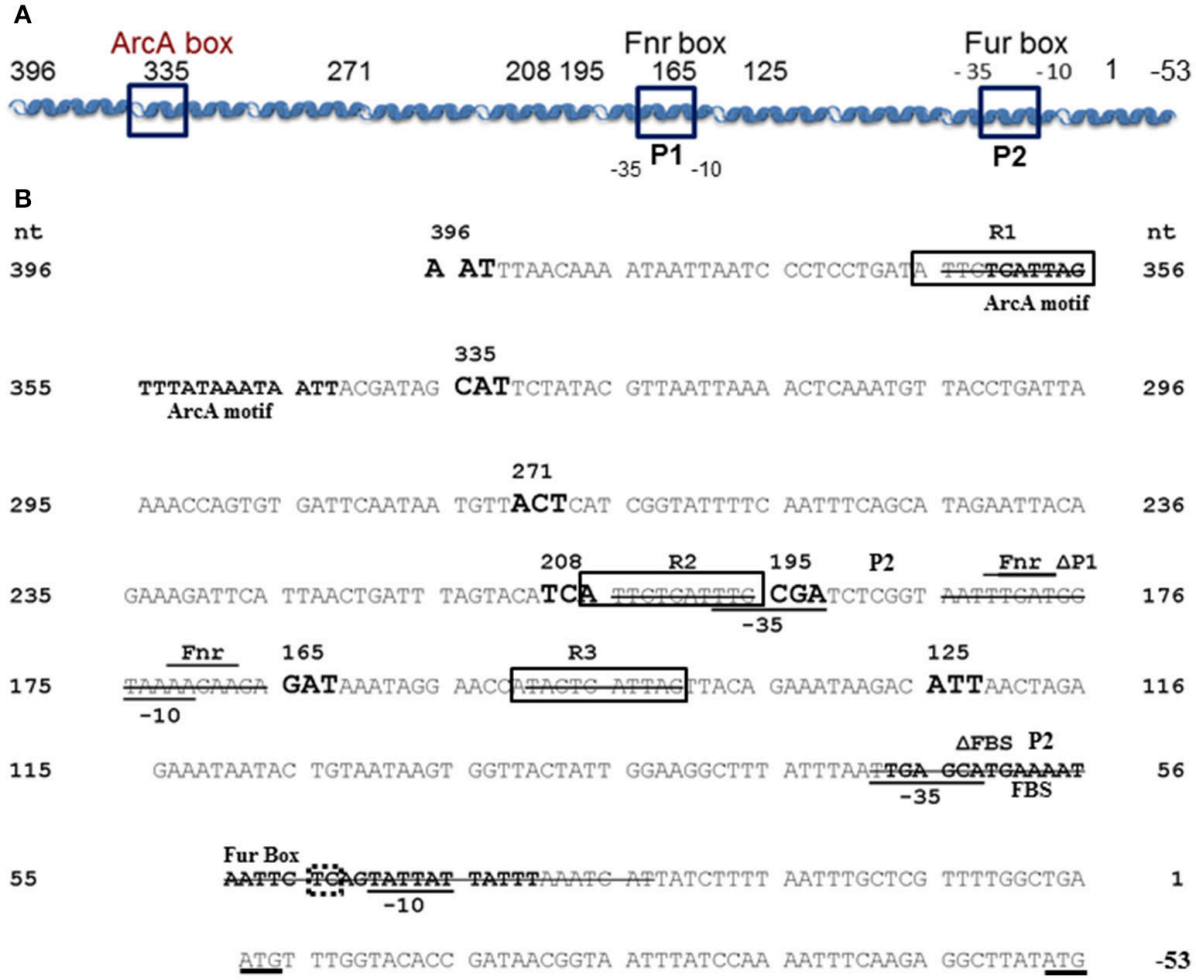
*Y. pestis feoABC* promoter region. **(A)** A diagram of the cloned *feoABC* promoter region showing two −10 −35 regions (P1 and P2 promoters), the proven Fur binding site (FBS) and regions with the best match to Fnr and ArcA motifs. The size and locations of 5′-truncations cloned into *lacZ* reporters are numbered and all except a 447 bp promoter fragment have 3′ ends at nucleotide (nt) 1; the *feoA*447 promoter fragment spans nt 396 to −53. **(B)** The sequence of the cloned region is shown with nt 1 corresponding to the first nt upstream of the original annotated translational start for *feoA*. A transcriptional stop is upstream of nt 396 and nt −51 to −50 corresponds to an updated annotated translational start for *feoA*. −35 and −10 regions are underlined as are the two different annotated ATG starts. Three repeats (R1–R3) are boxed as is the FBS. Various deletions in reporters have a line through the deleted nucleotide sequences. The FBS and the best match to a predicted ArcA motif in *Shigella* (Boulette and Payne, 2007) are labeled and in bold font. The over lined and labeled residues indicate the best match for a putative Fnr binding site consensus. The various 5′-truncations cloned into *lacZ* reporters are numbered and shown in increased, bold font. With one exception, the promoter regions for all reporter constructions end with nt 1. The dotted box around TC residues of the Fur-repressible P2 promoter element indicate the −13 and −14 residues indicative of a σ^S^-dependent promoter.

Primer pair Ecfeo-5′ and Ecfeo-3′ was used to amplify an ~329 bp fragment from DH5α. The 3′ end of this fragment corresponds to the equivalent of nucleotide 1 in Figure 1 (just prior to the predicted ATG start at 0 to −2). The PCR fragment was digested with *Pme*I and *Asc*I and cloned into the same sites of pWSK*feoA*::*lacZ*. Clones containing the correct insert were identified using Ecfeo-5′ and the universal −40 primer, verified by sequencing, and one clone (pWSK*feoA*_*E*.*coli*_::*lacZ*) was electroporated into KIM6+ and KIM6-2030.

#### Construction of the integration vector pUC18R6K-lac-Gm

To construct a pUC18R6K-mini-Tn*7*T-Gm integration vector containing *lacZ* and a multi-cloning site (MCS), we first moved the *lacZ* gene with the *feo* promoter from pBSfeoPlacZΔRs (Fetherston et al., 2012) into pWSK129. The plasmid, pBSfeoPlacZΔRs, was digested with *Eag*I and filled in with Klenow. The ~3.8 Kb fragment containing *lacZ* and the *feo* promoter was cloned into the *Eco*RV site of pWSK129 to generate pWSKfeoPlacZΔRs. This plasmid was digested with *Pme*I and *Asc*I to remove the *feo* promoter and a multiple cloning site (MCS) was inserted in its place. The MCS was isolated from a PCR reaction containing pNEB193 as a template with the universal −40 and reverse universal primers. The PCR product was digested with *Pme*I and *Asc*I and cloned into the *Pme*I/*Asc*I sites of pWSKfeoPlacZΔRs to generate pWSKlacMCS. The presence of the MCS was confirmed by sequencing. An ~3.5 Kb *Xho*I/*Xma*I fragment containing the *lac*Z gene with the MCS was isolated from pWSKlacMCS and cloned into the *Xho*I and *Xma*I sites of pUC18R6K-mini-Tn*7*T-Gm (Choi et al., 2005) to generate the integration vector, pUC18R6K-lac-Gm. A clone containing the correct insertion was identified by PCR with the universal −40 primer and integvec-up.

#### Cloning *feoA, feoA165, feoA208*, and *feoA447* promoters into pUC18R6K-lac-Gm

A *Pme*I/*Xho*I fragment containing *lac*Z with the respective *feo* promoter was isolated from pWSKfeoPlacZΔRs, pWSKfeoA165::lacZ as well as pWSKfeoA208::lacZ and ligated to the 3.4 Kb *Pme*I/*Xho*I fragment of pUC18R6K-lac-Gm to yield pUCR6KfeoA-lac-Gm, pUCfeoA165-lac-Gm and pUCR6Kfeo208-lac-Gm. Plasmids with the correct configuration were identified by PCR using primers Feo-Pro2 and integvec-up as well as by restriction enzyme digests. Following electroporation into KIM6+ along with pTNS2, strains containing the appropriate sequences integrated at the Tn*7* insertion site were identified by PCR with primer pair Feo-Pro2 and attTn*7*Yp-fwd and designated KIM6-2203+ (*att*Tn*7:: feoA::lacZ*), KIM6-2204+ (*att*Tn*7:: feoA165::lacZ*) and KIM6-2205+ (*att*Tn*7:: feoA208::lacZ*).

The *feo*A447 promoter fragment (spanning nucleotides 396 to −50 in Figure 1) was isolated from a PCR reaction containing pWSKfeo (Fetherston et al., 2012) as a template with primers Feo-Pro1 and FeoP-4. The PCR products were eluted from Zymo columns, digested with *Pme*I and *Asc*I, and ligated into the same sites in pNEB193. A plasmid containing the *feo*A447 fragment was identified by sequence analysis and termed pNEBfeoP447. The *Pme*I/*Asc*I promoter fragment from pNEBfeoP447 was then ligated into the *Pme*I/*Asc*I sites of pUCR6K-lac-Gm. The resulting clones were screened using primers FeoP-4 and integvec-up as well as by restriction enzyme digests to identify a pUCR6K derivative carrying *lac*Z with the *feo*A447 promoter. This plasmid (pUCR6KfeoP447-lac-Gm) was introduced into KIM6+ along with pTNS2 and screened for integration by PCR with Feo-Pro2 and attTn*7*Yp-fwd primers. A strain containing the proper integrated reported was designated KIM6-2206+ (*att*Tn*7:: feoA447::lacZ*).

#### Deletion of specific sequences within the *feo* promoter of *Y. pestis*

Twenty base pairs encompassing the putative upstream (P1) promoter region of the *feo* operon were deleted using primer pair dfeoP1-5 and dfeoP1-3 in an overlapping PCR reaction with pNEBfeoP DNA as a template and Phusion (NEB). The PCR products were cleaned through Zymo columns, digested with *Dpn*I, and transformed into DH5α. Plasmids containing the deletion were detected by PCR using primers pfeo2 and pfeo-R6. The products were analyzed on 5% polyacrylamide gels to detect the 20 bp deletion which was confirmed by sequencing. A *Pme*I/*Asc*I fragment was isolated from one of the clones (designated pNEBΔfeoP1) that contained the correct sequence and inserted into the corresponding sites of pUCR6K-lac-Gm. The ligation reactions were transformed into DH5α (λ pir) and plated on LB agar with 100 μg of ampicillin/ml. Plasmids containing an insert were identified by PCR using primers Feo-Pro2 and integvec-up. One of these, designated pUCR6K-ΔfeoP1-lac-Gm, was electroporated along with pTNS2 into KIM6+, incubated for 1 h at 30°C and plated on TBA containing 10 μg of gentamicin/ml. A strain in which the *feo lac* construct was integrated into the Tn*7* insertion site was identified by PCR using primers Feo-Pro2 and attTn*7*Yp-fwd and designated KIM6-2207+ (*att*Tn*7:: feoA*Δ*feoP1::lacZ*).

The same basic procedure was used to delete the P2 promoter region [41 bps which include the Fur binding site (FBS), −10 and −35 regions; Gao et al., 2008] and three repeated sequences within the *feo* promoter region (R1-3; Figure 1). Primers dfeoFBS-3 and dfeoFBS-5 were used to delete the P2 promoter region. Initial clones (pNEBΔfeoFBS) were screened with primer pair Feo-Pro2 and feoΔ20-5. Plasmids containing an insert in pUCR6K-lac-Gm were identified using integvec-up and the universal −40 primer and designated pUCR6K-ΔfeoFBS-lac-Gm.

Primer pairs FeoR1d-5 and FeoR1d-3, FeoR2d-5 and FeoR2d-3, and FeoΔR3-5 with FeoΔR3-3 were used to delete 10 bp that included repeated sequences R1, R2, and R3 (Figure 1) in separate PCR reactions. The pNEB clones with deletions of R2 and R3, designated pNEBfeoΔR2 and pNEBfeoΔR3 respectively, were initially identified by PCR with primer pair pfeo2 and pfeo-R6. Clones with deletions of R1, termed pNEBfeoΔR1, were screened with primers Feo-Pro1 and FeoR2d-5. The final constructs in the integration vector were named pUCR6KfeoΔR1, pUCR6KfeoΔR2, and pUCR6KfeoΔR3.

Suicide plasmids with the promoter deletions were separately electroporated along with pTNS2 into KIM6+, incubated for 1 h at 30°C and plated on TBA containing 10 μg of gentamicin/ml. A strain in which the *feo lacZ* reporter deletion was integrated into the Tn*7* insertion site was identified by PCR using primers Feo-Pro2 and attTn*7*Yp-fwd. These strains were designated KIM6-2208+ (*att*Tn*7:: feoA*Δ*FBS::lacZ*), KIM6-2209+ (*att*Tn*7:: feoA*Δ*R1::lacZ*), KIM6-2210+ (*att*Tn*7:: feoA*Δ*R2::lacZ*), and KIM6-2211+ (*att*Tn*7:: feoA*Δ*R3::lacZ*).

### β-galactosidase assays

From TBA slants, *Y. pestis* cells were inoculated to an OD_620_ of 0.1 in chelex 100-treated PMH2 (cPMH2) at a pH of 6.0, 6.5, or 7.5 with or without 10 μM FeCl_3_ and grown at 37°C at 250 rpm through 2 transfers (6-8 generations). Lysates were assayed for enzymatic activity using ONPG (4-nitrophenyl-β-D-galactopyranoside) as a substrate as previously described (Miller, 1992). The statistical significances of differences were calculated using the 2-tailed student's *t*-test.

### Iron transport studies

*Y. pestis* cells were grown in cPMH2 under aerobic conditions for ~5 generations prior to use in iron transport assays under reducing conditions. Transport was initiated by adding ^55^FeCl_3_ to a final concentration of 0.2 μCi/ml to cultures containing 5 mM sodium ascorbate. Parallel cultures, preincubated for 10 min with 100 μM carbonyl cyanide *m*-chlorophenylhydrazone (CCCP), were used to demonstrate energy-independent binding. The ^55^FeCl_3_ used in the transport studies was diluted into PMH2 containing 100 mM sodium ascorbate, incubated for at least 30 min at room temperature, and filtered through a 0.45-μm filter before being added to the cultures. Duplicate samples (0.5 ml) were taken at various time points after the addition of ^55^FeCl_3_, filtered through 0.45-μm GN-6 nitrocellulose membranes, and rinsed twice with PMH2 medium containing 20 μM FeCl_3_. The membranes were added to vials containing Bio-Safe II scintillation fluid (Research Products International) and counted in a Beckman LS3801 liquid scintillation counter. Unfiltered samples were used to determine the total amount of radioactivity in each culture. The results are expressed as percent uptake/0.4 OD_620_ to compensate for slight increases in cell density during the course of the assay.

For transport under non-reducing conditions *Y. pestis* cells were grown in cPMH1 for ~5 generations prior to use in iron transport assays. Transport was initiated by adding filtered ^55^FeCl_3_ to a final concentration of 0.2 μCi/ml to cultures containing cold FeCl_3._ Cultures were processed and analyzed as described above.

### Electrophoretic mobility shift assays

Electrophoretic mobility shift assays (EMSAs) were used to demonstrate binding to the *feo* promoter region. Full length and truncated *feo* promoter probes were amplified by PCR using primer pairs pfeoF4 and pfeoR4, pfeoF5 and pfeoR5, or pfeoF6 and pFeoR6 (Table S2). *Y. pestis* KIM6+ or KIM6 *fur::kan-9* (KIM6-2030) were grown with or without added iron in cPMH2 media through 2 passages to an OD_620_ of 0.3–0.4, cells centrifuged and frozen at −80°C. Cell pellets were resuspended in 2 ml of B-PER II reagent, 2 μl of lysozyme (10 mg/μl), 2 μL of DNase I and 4 μL of DNase I buffer and incubated with shaking for 20 min at room temperature. Samples were centrifuged to remove debris and protein content analyzed by the Bradford assay and comparison by SDS-PAGE. Lysates were aliquoted and stored at 4°C. Binding reactions [100 mM Tris, 750 mM KCl, 10 mM dithiothreitol (DTT), 50 mM MgCl_2_, 12.5% glycerol, 50 mM EDTA [pH 8], 20 fmol biotinylated DNA probe, and *Y. pestis* cell lysates] were incubated at room temperature for 20 min. 5X EMSA loading buffer (50 mM Tris, 250 mM KCl, 5 mM DTT, 37 μM bromophenol blue) was added and the reactions were loaded onto a 6% DNA retardation gel (Invitrogen) with prechilled 0.5X TBE buffer and run at a constant voltage of 120 V for 90 min. After electrophoresis, gels were transferred to a Biodyne B Nylon membrane (Thermo Fisher Scientific) with prechilled 0.5X TBE buffer at a constant voltatge of 120 V for 45 min. Following transfer, the membrane was crosslinked with a UV Stratalinker 2400 (Stratagene, La Jolla, CA) and incubated overnight with 20 mL blocking buffer (Thermo Fisher Scientific). The Chemiluminescent Nucleic Acid Detection Module (Thermo Fisher Scientific) was used according to the manufacturer's protocol. The membrane was visualized on CL-X Posure Film (Thermo Fisher Scientific).

## Results

### Truncations of the *feoA* promoter affect overall expression and repression by Fe-Fur

**Figure 2:**
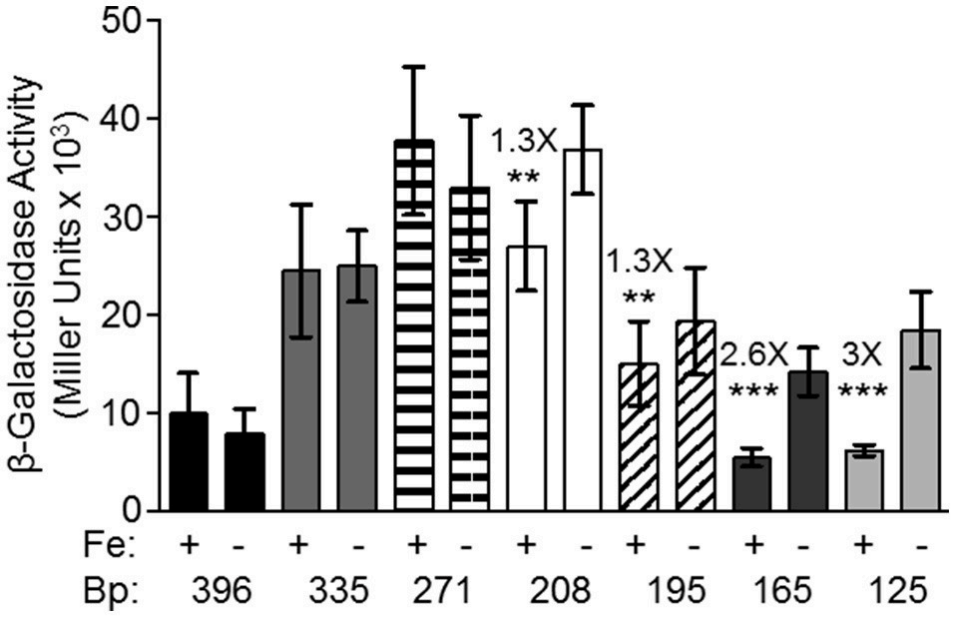
The 396 bp *feoABC* promoter is not repressed by iron under aerobic conditions, while the 165 and 125 bp truncated promoters are repressed by iron. KIM6+ carrying the various reporter plasmids were grown in the presence or absence of 10 μM FeCl_3_ at 37°C under aerobic conditions in cPMH2. Samples were taken during mid-exponential phase for β-galactosidase assays. Activities are averages of multiple samples from at least two independent experiments expressed in Miller units. Error bars represent standard deviations. Statistically significant fold differences due to iron status are shown (^**^P ≤ 0.003, ^***^P ≤ 0.0002).

Although, all other tested promoters controlling expression of iron transport systems in *Y. pestis*, are repressed by iron under aerobic conditions, the *feoA* promoter is repressed only under static (microaerobic) conditions. The other Fe^2+^ transporters (Yfe, Fet-flp, and Efe) are repressed by iron under borth aerobic and microaerobic conditionas and none of the 4 *Y. pestis* Fe^2+^ transporters are repressed by aerobic conditions compared to microaerobic conditions (Perry and Fetherston, 2004; Fetherston et al., 2012; Perry et al., 2012). We hypothesized that some aspect of the *feoA* promoter is preventing Fe-Fur regulation under aerobic conditions. To test this hypothesis we made 5′-truncations of the *feo* promoter region and fused them to a *lacZ* transcriptional reporter (Figure 1). KIM6 (Δ*pgm* Feo^+^) strains carrying the various reporter plasmids were grown in cPMH2 in the presence and absence of iron under aerobic growth conditions at 37°C. When the intergenic region between *y3913* and *feoA* is truncated from 396 to 335 bp, transcriptional activity increased ~2.5-fold in the presence and absence of iron (Figure 2). This activity was further increased to a 4-fold difference, both in the presence and absence of iron when the 396 bp promoter region was truncated to 271 bp. A modest but statistically significant 1.3-fold ron-mediated repression was not observed until the *feoA* promoter region was truncated to 208 bp. This construct still had overall increased transcriptional activity compared to the full-length 396 bp promoter region (Figure 2). A further truncation to 195 bp maintained repression in the presence of iron, while the overall transcriptional activity decreased. The 165 and 125 bp promoters, showed stronger iron-mediated repression (2.6- and 3-fold, respectively), and overall transcriptional activity that returned to the 396bp promoter levels (Figure 2). Thus, the 396 to 165 bp region somewhat prevents Fe-Fur repression aerobically, while the 396 to 335 bp region somewhat lowers overall transcriptional activity from the *feoA* promoter region.

### The *feoA* promoter is iron-repressed aerobically under acidic conditions

In *Salmonella*, RstA activates transcription from the *feo* promoter (Jeon et al., 2008). Previous work showed that a Δ*rstAB* mutation does not affect transcription from the *Y. pestis feoA* promoter (Fetherston et al., 2012). Since the *rstAB* operon is activated by the PhoP/PhoQ two-component regulators, which are activated by acidic pH (Jeon et al., 2008; Choi et al., 2009), we examined whether acidic conditions affected transcription from the *feoA* promoter. *Y. pestis* cells with full-length or truncated promoters were cultured in cPMH2 at pHs of 6.0, 6.5, or 7.5. It should be noted that all of the strains grew very slowly at pH 6.

**Figure 3:**
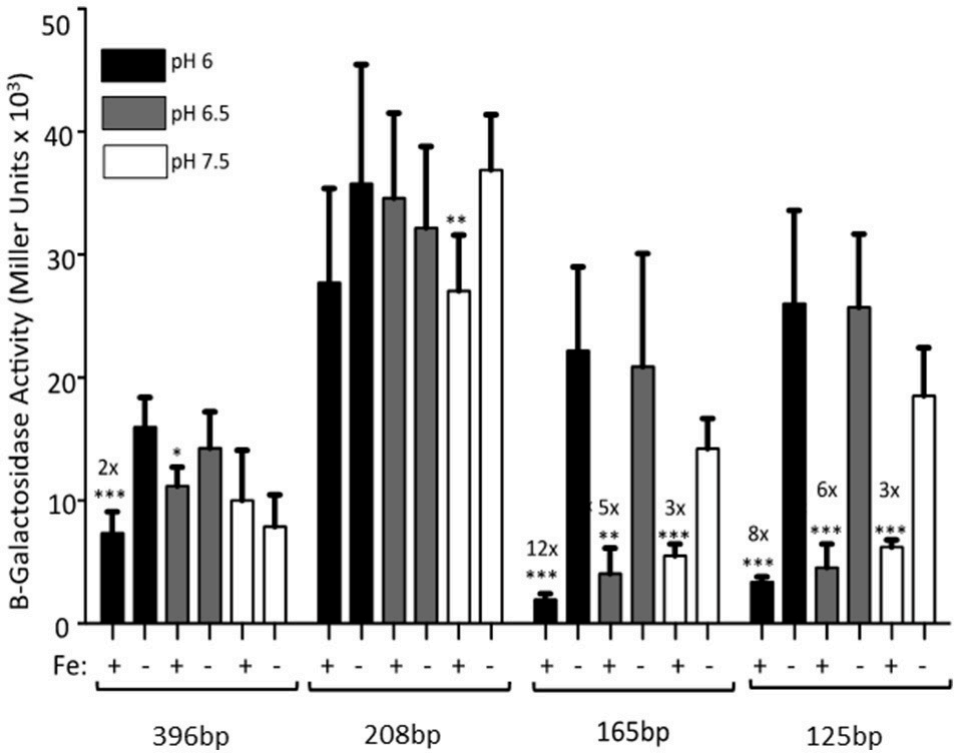
The full-length *feoABC* promoter is repressed aerobically by iron under acidic conditions. KIM6 carrying the various reporter plasmids were grown in the presence or absence of 10 μM FeCl_3_ at 37°C under aerobic conditions in cPMH2 at pH 6, pH 6.5, or pH 7.5. Samples were taken during mid-exponential phase for β-galactosidase assays. Activities are averages of multiple samples from at least two independent experiments expressed in Miller units. Error bars represent standard deviations. Statistically significant fold differences due to iron status are shown (^*^P ≤ 0.02, ^**^P ≤ 0.002, ^***^P ≤ 0.0002).

The 396 bp promoter had a 2-fold repression in the presence of iron when the cultures were grown at pH 6. A slight, but not statistically significant repression was seen at pH 6.5. Expression levels at pH 7.5 (from Figure 2) are shown for all constructs for reference (Figure 3). However, RstAB is not responsible for this effect since KIM6+ (*rstAB*^+^) and KIM6-2189+ (Δ*rstAB*) had similar levels of activity from the *feoA::lacZ* reporter (Fetherston et al., 2012).

When the promoter was truncated to 208 bp, there was no significant iron repression at any of the tested pH levels (Figure 3), perhaps due to the high standard deviations associated with this reporter. While the 165 and 125 bp promoter truncations, showed ~3-fold iron-mediated repression at pH 7.5, iron repression increased to 5- and 6-fold at pH 6.5, respectively. When the pH was further lowered to 6, the iron repression became even more pronounced—12- and 8-fold respectively. At all pHs, the pattern of increased expression from the 208 bp reporter, compared to the 396, 165, and 125 bp reporters was maintained (Figure 3).

### Iron repression of the truncated *feo* promoters is dependent on fur

**Figure 4:**
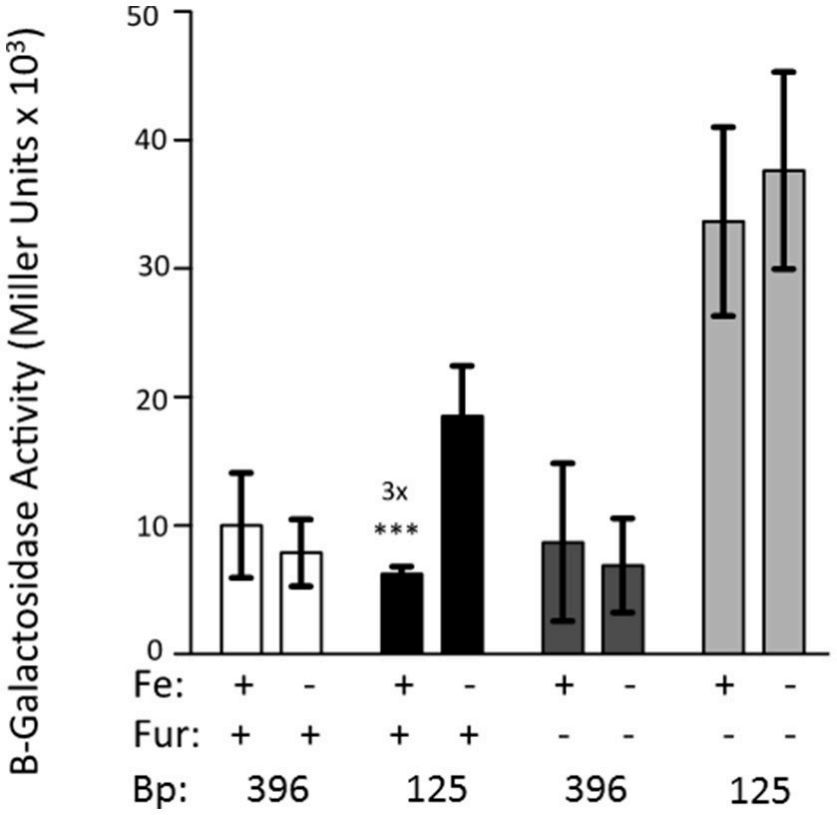
Fur is necessary for the iron-repression of the 125 bp promoter clone. KIM6 (*fur*^+^) and KIM6-2030 (*fur::kan*), carrying either the 396 or 125 bp reporter plasmids were grown in the presence or absence of 10 μM FeCl_3_ at 37°C under aerobic conditions in cPMH2. Samples were taken during mid-exponential phase for β-galactosidase assays. Activities are averages of multiple samples from at least two independent experiments expressed in Miller units. Error bars represent standard deviations. The statistically significant fold difference due to iron status is shown (^***^P < 0.0002).

The 396 bp reporter does not exhibit iron repression. However, truncation of the promoter region to 125 bp causes transcriptional repression of the reporter by iron (Figure 2). This experiment was repeated using KIM6-2030 (*fur::kan* mutant) carrying the 396 or 125 bp reporter. With this *fur* mutant the 125 bp promoter reporter no longer exhibited iron repression. This indicates that Fur is responsible for the observed repression of the 125 bp reporter (Figure 4).

### The lack of iron-repression of the *feo* promoter is due to the promoter sequence, not a property of the *Y. pestis* fur protein

To determine whether the *feo* promoter itself or a unique aspect of the Fur protein in *Y. pestis* was preventing aerobic iron repression of the *feoA* promoter, we used a *lacZ* transcriptional reporter fused to the *E. coli feo* promoter region. The *E. coli feo::lacZ* reporter showed iron repression under aerobic conditions in *Y. pestis* (Figure S1). This suggests that a unique aspect of the *Y. pestis* Fur protein is not responsible for the lack of iron repression of the *Y. pestis feo* promoter under aerobic conditions. Rather the sequence/structure of the *Y. pestis feoA* promoter region likely prevents iron repression under aerobic, but not microaerobic conditions.

### A protein(s) that is not fur is binding to the *feo* promoter region

**Figure 5:**
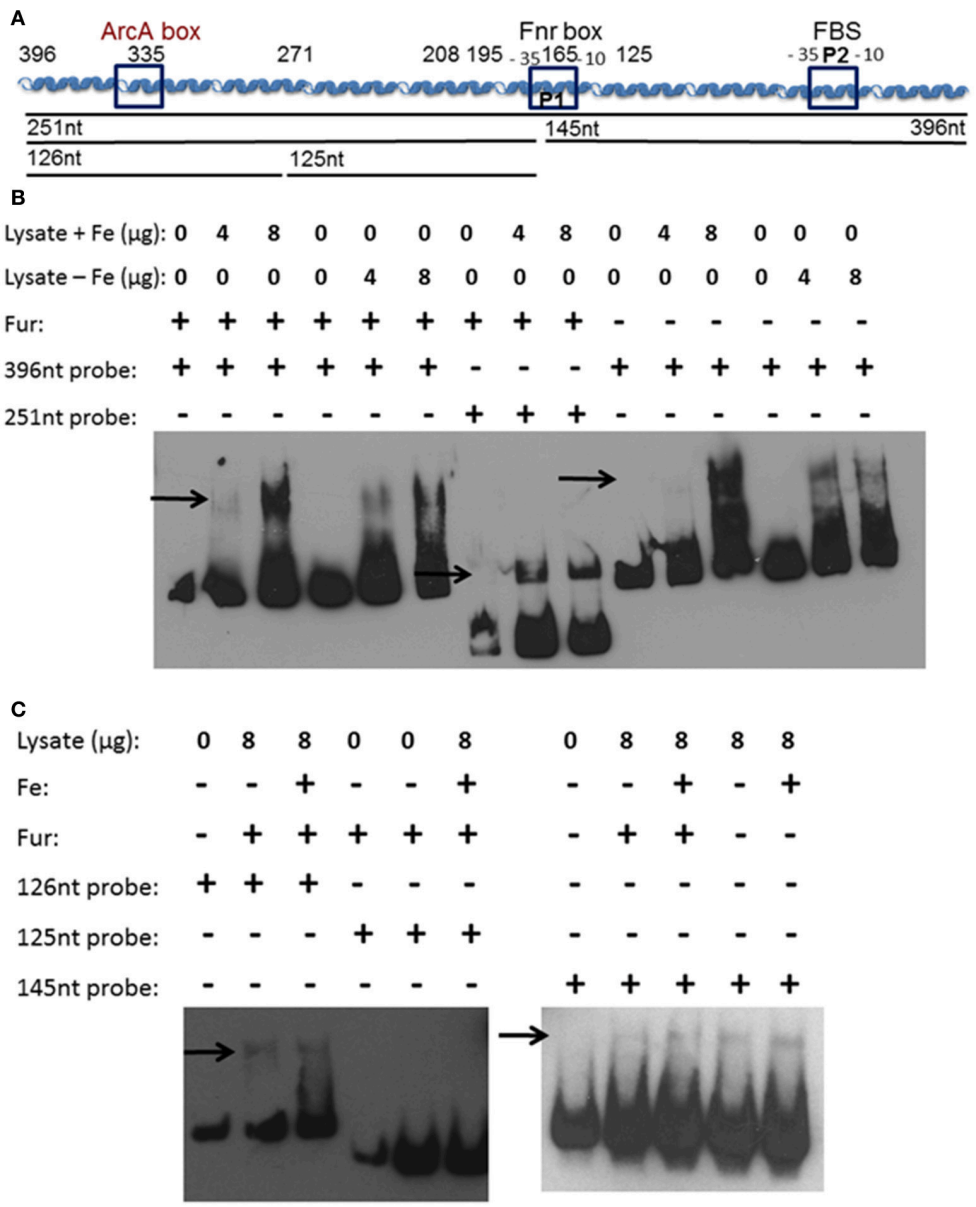
A soluble protein(s) bind(s) the *feoA* promoter in at least two locations. **(A)** A diagram of the cloned *feoABC* promoter region showing two −10 −35 regions (P1 and P2), the proven FBS and regions with the best match to Fnr and ArcA motifs. The size and locations of 5′-truncations cloned into *lacZ* reporters are numbered. The DNA probes used in EMSAs are depicted as black lines with the nucleotide size indicated. **(B)** EMSAs using KIM6+ (*fur*^+^) or KIM6-2030 (*fur::kan*) whole cell lysates with or without added iron and the 396 or 251 bp biotinylated probes. **(C)** EMSAs using KIM6+ or KIM6-2030 whole cell lysates with or without added iron and the 126 bp, 125 bp or 145 biotinylated probes. Representative blots from two or more independent analyses are shown. Arrows indicate mobility-shifted bands.

We hypothesized that a repressor binding to the *feo* promoter region between 396 and 208 bp is the reason for increased transcriptional activity in the truncated promoters. Additionally, a second protein binding to the *feoA* promoter may prevent iron-repression under aerobic conditions. To examine these possibilities, an electrophoretic mobility shift assay (EMSA) was performed using whole cell lysates from *Y. pestis* that had been grown aerobically in the presence or absence of iron. Biotinylated probes were generated by PCR using primers that spanned the 396 bp *feoA* promoter, a 251 bp 3′-truncated *feo* promoter fragment, or a 146 bp 5′-truncated *feo* promoter fragment (Figure 5A). A shift was observed using the 396 bp (Figure 5B), 251 bp (Figure 5B), and 126 bp (Figure 5C) probes regardless of growth conditions.

We would not expect to see Fur binding in the EMSAs since Fur needs to be bound to a divalent cation in order to bind DNA (de Lorenzo et al., 1988). Even in the cultures where iron was added, the intracellular iron bound to Fur would likely be converted in the ferric form after cell lysis. Typically MnCl_2_ is added to cell lysates or purified Fur, when studying Fur binding, since the Mn^2+^ serves as a stable divalent cation under aerobic conditions (Friedman and O'Brian, 2003). However, since there is a Fur-binding site in the *feoA* promoter it was necessary to ensure that Fur was not responsible for this shift. Cell lysates from the *fur* mutant grown with or without iron were also used, and also resulted in a shift with the 396 bp (Figure 5B) and 146 bp (Figure 5C) probes. The 251 bp probe was not tested. Therefore, this observed shift is not due to Fur binding, but some other *Y. pestis* protein. In order to further identify the region in which the protein(s) is binding, two additional biotinylated probes were generated by PCR, splitting the 251 bp probe into an upstream 126 bp probe and downstream 125 bp probe (Figure 5A). Regardless of growth condition, a shift was seen with the upstream 126 bp probe, but not with the 125 bp downstream probe (Figure 5C). These data suggest that there are (1) two proteins binding the *feo* promoter: one within the 126 bp probe (396 to 271 bp region in Figure 1) and one within the 146 bp probe (146 to 1 bp region in Figure 1) or (2) one protein binding to both regions.

### The lack of aerobic iron regulation is not due to titration of a transcriptional regulator

Although, the pWSK vectors are considered low-copy number (Wang and Kushner, 1991) which we have used in transcriptional analysis of transcriptional regulation of numerous promoters (Fetherston et al., 1999, 2012; Gong et al., 2001; Kirillina et al., 2006; Forman et al., 2010; Perry et al., 2012), we wanted to ensure that our results were not due to titration of some negative regulatory factor by the *feoA* reporter plasmids. Therefore, we constructed vectors where the reporter gene would integrate into the Tn*7* insertion site of *Y. pestis* using the Tn*7*-based system developed by Choi et al. (2005). We tested integrated reporters with the *feoA* (396 bp), *feoA165*, and *feoA208 lacZ* reporters in KIM6+ grown under static and aerobic conditions in the presence or absence of iron. The integrated reporters behaved in the same fashion as their plasmid counterparts (compare Figure S2 and Figure 2) with regard to the overall transcriptional activity and iron regulation. Thus, the *feoA* construct was only repressed by iron in cultures grown under static conditions while *feo*A165 reporter was iron-regulated under both static and aerobic conditions. The activity of the integrated *feo*A208::*lac*Z construct was much higher than the other two *feo* constructs and exhibited the same regulation pattern as its plasmid counterpart (compare Figure S2 and Figure 2). The integrated *lac*Z gene without any promoter fragment exhibited very low activity under all conditions (Figure S2).

We also used the integrated reporter system to examine regulation of an extended fragment of the *feoA* promoter region. Subsequent analysis and annotation of the *Y. pestis feoABC* operon predicts the start codon for *feo*A is further downstream than the originally annotated initiating ATG (at 0 to −2) in Figure 1. To determine if the additional bp separating the 2 predicted ATG start codons would affect the regulation of the *feo* operon, we made another *feoA*::*lacZ* reporter (pUCR6KfeoP447-lac-Gm) that included these nucleotides and integrated it into the Tn*7* insertion site of KIM6+. The reporter with the extended promoter fragment exhibited the same properties as our original *feoA*::*lacZ* construct (data not shown) indicating that the additional base pairs did not play a role in the regulation of the *feoABC* operon.

### The *feoABC* locus is likely transcribed from two independent promoters

**Figure 6:**
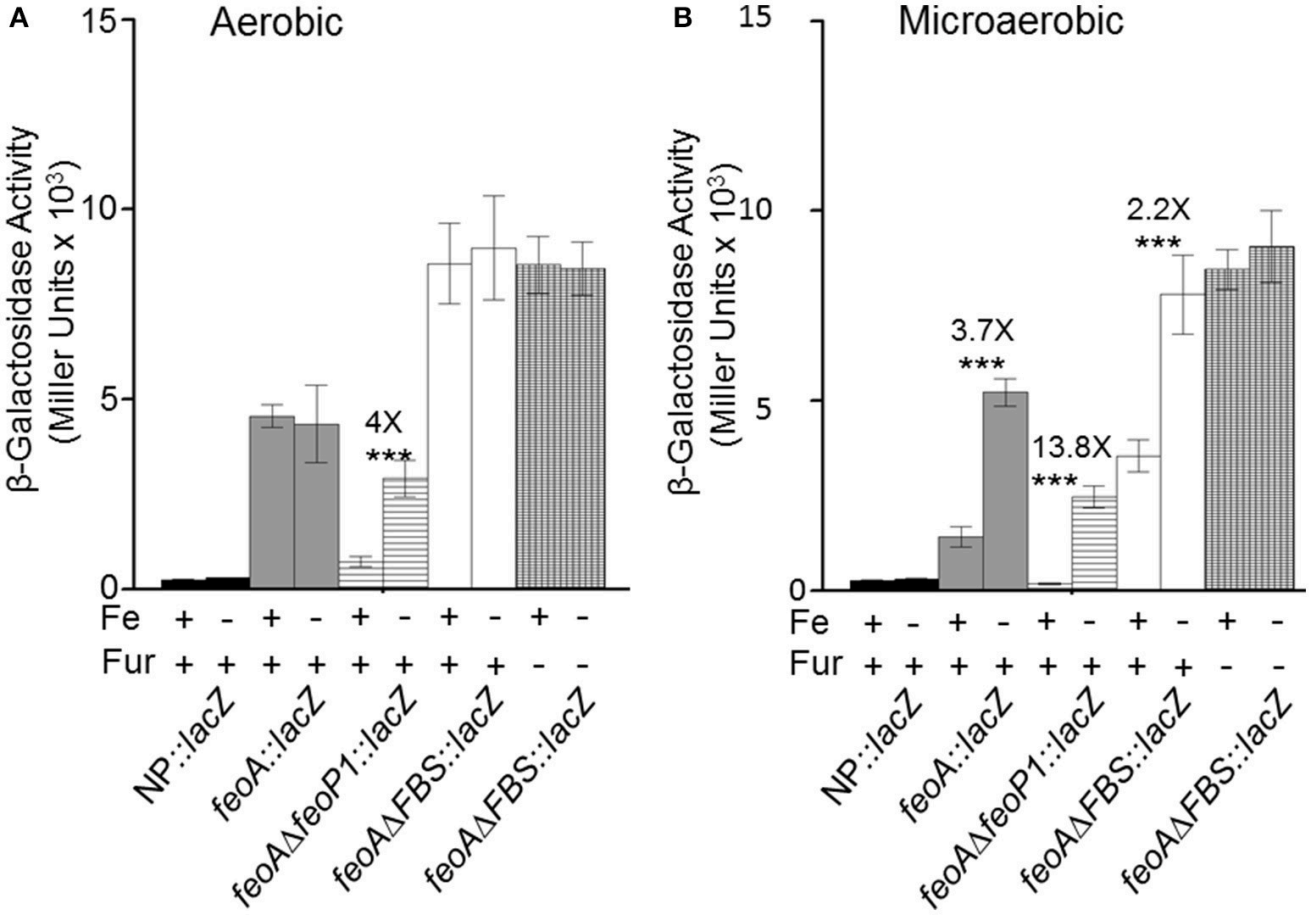
Transcription from integrated *feoA* promoters occurs in the presence and absence of P1 or P2 promoter regions aerobically and microaerobically. Transcription from the *att*Tn*7*::*feoA*::*lacZ* reporter (KIM6-2202+) is compared to transcription from the *attTn7::feoA*Δ*P1::lacZ* (KIM6-2207+) and *attTn7::feoA*Δ*FBS::lacZ* (KIM6-2208+) reporters. The FBS deletion also deletes the −10 and −35 region of the P2 promoter. KIM6-2212+ (*att*Tn*7::lacZ*; no promoter region) is used as a negative control. All strains were grown in the presence or absence of 10 μM FeCl_3_ at 37°C under aerobic or microaerobic conditions in cPMH2. Samples were taken during mid-exponential phase for β-galactosidase assays. Activities are averages of multiple samples from at least two independent experiments expressed in Miller units. Error bars represent standard deviation. The statistically significant fold difference due to iron status is shown (^***^P < 0.0002).

The transcriptional and binding data caused us to re-examine the *feoA* promoter region. Another, putative RNA polymerase (RNAP)—binding site (labeled P1/-10/-35 in Figure 1; the putative P1 promoter spans the Fnr binding site) is located upstream of the FBS and the P2 promoter (Figure 1). To test this, we deleted 41 bp that encompass the FBS and includes the putative −35 and −10 regions for the downstream P2 promoter. This strain, designated KIM6-2208+ (*att*Tn*7:: feoA*Δ*FBS::lacZ*) had higher β-galactosidase activity (1.5–2.5X) than the integrated reporter with the original *feoA*::lac*Z* construct (KIM6-2203+; *att*Tn*7:: feoA::lacZ)* under all conditions (Figure 6). Absence of the downstream P1 promoter may allow better read through from transcription initiation at the upstream P2 promoter. Interestingly, the *att*Tn*7:: feoA*Δ*FBS::lacZ*) reporter was slightly repressed (~2-fold) by iron under static but not aerobic conditions. Examination of the *feoA* promoter revealed additional potential FBSs with weaker matches to the *Y. pestis* consensus FBS (Zhou et al., 2006), located at bp 262–280 (11/19 bp) and bp 204–222 (12/19). Whether, either of these regions is responsible for the iron repression observed with the *att*Tn*7:: feoA*Δ*FBS::lacZ* reporter is unknown; however, the observed ~2-fold iron repression was alleviated in a *Y. pestis fur* mutant (KIM6-2030+; Figure 6).

Deletion of 20 bp that would include the putative −10 region of the upstream P1 promoter resulted in an integrated construct (*att*Tn*7:: feoA*Δ*feoP1::lacZ* in KIM6-2207+) with lower overall β-galactosidase activity than the original *att*Tn*7:: feoA::lacZ* reporter in KIM6-2203+ under both static and aerobic conditions. However, like the *att*Tn*7:: feoA165::lacZ* reporter in KIM6-2204+, the *att*Tn*7:: feoA*Δ*feoP1::lacZ* reporter was repressed by iron under both conditions. This provides additional evidence that the *Y. pestis feo* operon contains 2 promoter regions with the downstream P2 promoter being repressed by iron when cells are grown either statically or aerobically and suggest that the upstream P1 promoter is more active and slightly repressed by iron only under static conditions.

In examining the *Y. pestis feo* promoter region, we noted the presence of 3 nearly identical 11 bp direct repeat sequences, two of them located before the upstream P1 promoter (R1 and R2) and one (R3) present between the 2 promoters (Figure 1 and Table S3). To determine if these regions regulated expression of the *feoABC* operon, we deleted each of them in turn and analyzed the effect on the β -galactosidase activity of strains carrying the respective integrated reporters. An R3 deletion (*att*Tn*7:: feoA*Δ*R3::lacZ* in KIM6-2211+) had no significant effect on the expression of the *feoA*::*lacZ* reporter under any of the tested conditions. Similarly, deletion of R1, the most distally located repeat, had no effect on regulation in that the reporter construct (*att*Tn*7:: feoA*Δ*R1::lacZ* in KIM6-2209+) was only repressed by iron in cells grown under static conditions. Under aerobic conditions, the *att*Tn*7:: feoA*Δ*R1::lacZ* reporter was not repressed by iron but was less active (51–65%) than the *att*Tn*7:: feoA165::lacZ* reporter. The *att*Tn*7:: feoA*Δ*R2::lacZ* in KIM6-2211+ reporter in KIM6-2210+ behaved essentially the same as the *att*Tn*7:: feoA*Δ*feoP1::lacZ* reporter. Expression of the *lac*Z gene was lower relative to the *att*Tn*7:: feoA::lacZ* reporter but was repressed by iron under both static and aerobic growth conditions (Figure S3). The R2 deletion would remove a potential −35 box for the putative P1 promoter (Figure 1) which likely accounts for the results we obtained with this construct.

### The *FeoABC* and *YfeABCD* systems transport iron under both reducing and non-reducing conditions

**Figure 7:**
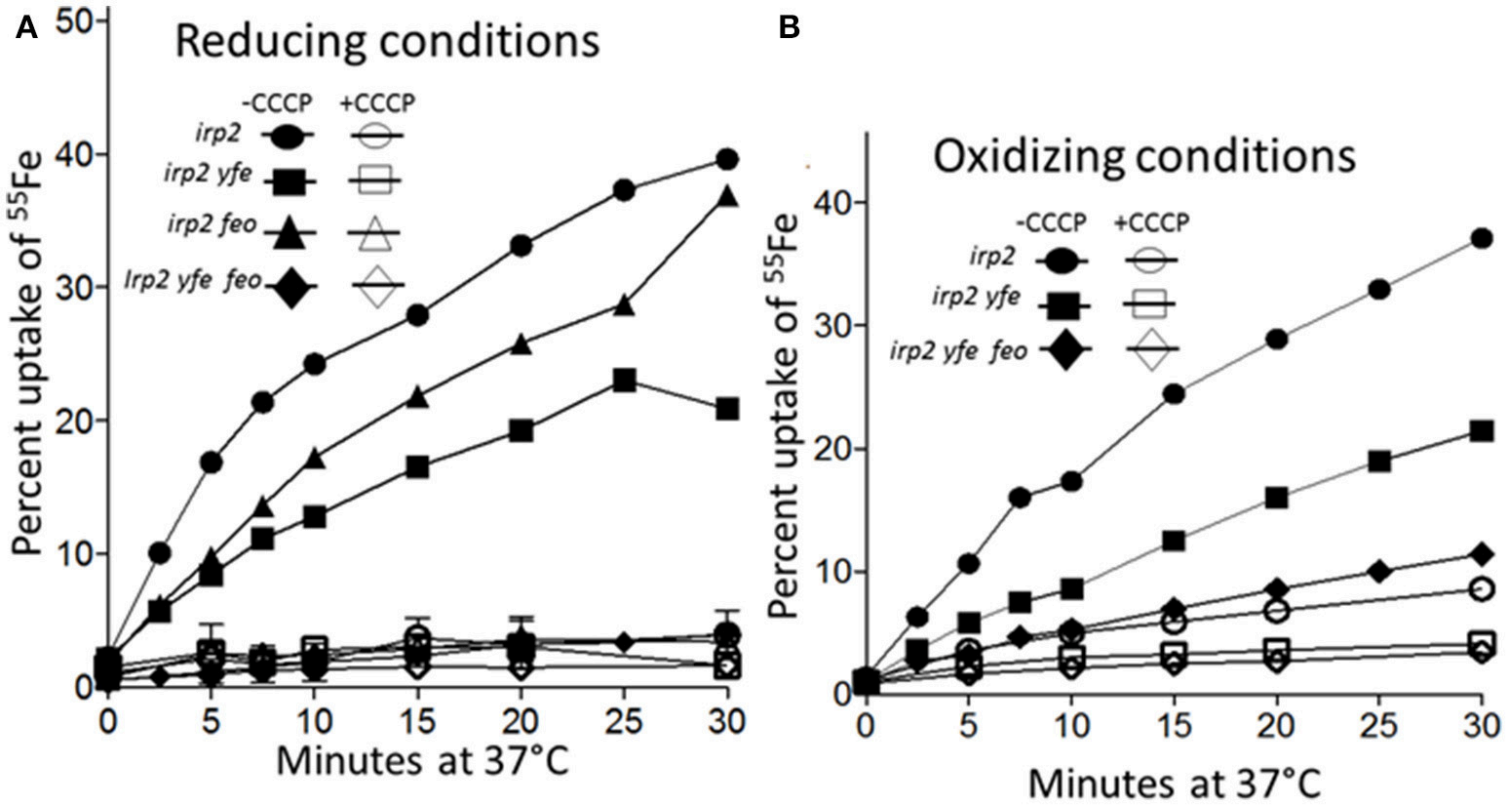
Iron uptake by the Feo and Yfe transporters under reducing **(A)** and non-reducing **(B)** conditions. The uptake of radiolabeled iron (^55^Fe) by *Y. pestis* strains Irp2^−^Yfe^+^ Feo^+^ [*irp2*; circles], Irp2^−^ Yfe^−^ Feo^+^ [*irp2 yfe*; squares], (Irp2^−^ Yfe^+^ Feo^−^[*irp2 feo*; triangles]), and (Irp2^−^ Yfe^−^ Feo^−^ [*irp2 yfe feo*; diamonds]) incubated at 37°C in the presence **(A)** or absence **(B)** of ascorbate was monitored over time. Cells were poisoned metabolically by incubation for 10 min with 100 μM CCCP before the addition of the isotope (open symbols). The uptake curves are averages of duplicate samples from two or more replicate experiments.

To demonstrate that the Yfe and Feo systems are involved in active iron transport, transport assays were performed under reducing and non-reducing conditions. Sodium ascorbate was utilized to maintain ^55^FeCl_3_ in the reduced, ferrous state. An Δ*irp2* mutant (unable to make Ybt), transported ~40% of the ferrous iron under reducing conditions (Figure 7A) and ~35% under non-reducing conditions (Figure 7B) by 30 min of incubation at 37°C. The uptake was energy-dependent, since CCCP-poisoned cells showed very little cell-associated iron (Figures 7A,B). The Δ*irp2* Δ*yfe* double mutant, transported iron at slightly reduced rates compared to the Δ*irp2* mutant, under both reducing (~3-fold) (Figure 7A) and non-reducing (~2-fold) (Figure 7B). The Δ*irp2* Δ*feo* double mutant had slightly reduced rates of uptake under reducing conditions compared to the Δ*irp2* mutant (Figure 7A). However, the Δ*irp2* Δ*yfe* Δ*feo* triple mutant showed very little cell-associated iron, a level comparable to the CCCP-poisoned cells, under both reducing (Figure 7A), and non-reducing (Figure 7B) conditions. This indicates, that Yfe and Feo have somewhat redundant *in vitro* ferrous uptake functions with one system partially compensating for loss of the other. However, together they are crucial for ferrous iron transport under the short-term *in vitro* uptake conditions used here. In addition, these assays indicate that the Yfe and Feo systems can use ferric iron as well as ferrous.

## Discussion

The FeoABC system is widespread in bacteria and transcriptional regulation by Fnr, ArcA, RstAB and Fur has been demonstrated, particularly in *E. coli, Salmonella*, and *Shigella* (Kammler et al., 1993; Boulette and Payne, 2007; Jeon et al., 2008). Although, the *Y. pestis feoA* promoter encodes putative putative Fnr- and ArcA-binding sites (Gao et al., 2008), Δ*fnr* and Δ*arcA* mutations do not affect *feoB* transcription. In addition, *oxyR, rstA*, or *feoC* mutations failed to affect *feoB* transcription. Finally, the *feoABC* operon is repressed by iron and Fur during microaerobic but not aerobic growth (Forman et al., 2010; Fetherston et al., 2012). Thus, the *Y. pestis feoABC* locus currently has unique regulatory properties.

To assess the mechanism behind the lack of iron and Fur repression during aerobic growth, we assessed the regulation of 5′-truncations of *feoA::lacZ* reporter with a 396 bp intergenic/promoter region. Modest aerobic iron-mediated repression occurred with the 208 bp *feoA::lacZ* and stronger aerobic iron- repression was observed with the 165 and 125 bp promoter truncations (Figure 2). This repression was increased when *Y. pestis* cells were grown at acidic pHs (6.0 and 6.5) and even the 396 bp *feoA*:*:lacZ* was repressed by iron after growth at pH 6.0 (Figure 3). When the *feoA* promoter from *E. coli* linked to *lacZ* was expressed in *Y. pestis*, it was repressed by iron aerobically (Figure S1), confirming that it is a unique aspect of the *Y. pestis feoA* promoter region that is preventing iron-Fur repression aerobically and not an aspect of the *Y. pestis* Fur protein. Thus, we propose that the 396 to 165 bp region somewhat prevents Fe-Fur repression aerobically and that pH has some influence on this.

In addition, when the promoter was truncated from 396 to 335 bp or 271 bp, there was an overall increase in transcriptional activity (Figure 2), suggesting that a repressor binds to the 396 to 335 bp region, lowering overall transcription. A series of EMSA experiments found that a *Y. pestis* cytoplasmic protein(s) bound to two regions: 396 to 271 and 145 to 1 bp (Figure 5). Although, we have not identified this protein(s) it is not Fur (Figure 5).

These results caused us to reexamine the sequence of the region upstream of *feoA*. This led to our hypothesis that a second promoter region (designated P1 in Figure 1; 198 to 171 bp which overlaps the putative Fnr-binding site) is upstream of the originally annotated Fur-repressible promoter (69 to 29 bp including the FBS; P2 in Figure 1). Note that the defined binding region for the 396 to 271 bp region lies outside of the predicted second promoter region. Significant transcriptional activity from the putative P2 promoter was demonstrated in a construct that deleted the P1 promoter region (*feoA*Δ*FBS::*lacZ). Iron addition showed a modest (~2-fold) repression of the P1 promoter under static (microaerobic) but not aerobic conditions. Although two weaker potential FBS sites (262–280 and 204–222) are upstream of the P1 promoter, their role in iron regulation has not been examined. We also identified three direct repeats (R1-R3 in Figure 1) and tested their role(s) on transcription of the *feoABC* operon. Deletion of R3 (151 to 141 bp region in Figure 1) had no effect on expression compared to the *feoA::lacZ* reporter, while the R1 (366 to 356 bp region in Figure 1) deletion reduced expression under aerobic conditions (51–65%) without altering iron regulation under either aerobic or microaerobic conditions (Figure S3). In contrast, the *feoA*Δ*R2::lacZ* had activity essentially the same as the *feoA*Δ*feoP1::lacZ* reporter. Note that the R2 repeat (206 to 196 bp region in Figure 1) is not within regions to which protein(s) bind (396 to 271 and 145 to 1 bp; Figure 5). Thus, the transcriptional effect observed in the R2 deletion likely occurs because this repeat overlaps the P1 −35 box (Figure 1 and Figure S3). Thus, there is no evidence that the R1-3 repeats play a direct role in transcriptional regulation from either P1 or P2.

In our current model, truncation of the 297 bp region to 125 or 165 bp, eliminates the upstream P1 promoter, forcing transcription from the second Fur-regulated P1 promoter (Figure 1). Although, the 195 and 208 bp promoter truncationss contained both RNAP-binding sites, binding would not be optimal since the RNAP typically spans −55 to +20. Thus, there may have been limited transcription from the upstream P1 promoter with the majority coming from the downstream iron-repressible P2 promoter. The upstream RNAP polymerase binding site may favor an alternative RNAP possibly with a σ^*S*^ subunit. In *E. coli*, Becker and Hengge-Aronis found that σ^*S*^-dependent promoters have a tendency to have a “C” at position −13 and either a “T” or “G” at position −14. Of the promoters they examined, 85% had a “C” at −13, 40% had a “T” at −14, and 40% had a “G” at −14 (Becker and Hengge-Aronis, 2001). The upstream RNA polymerase binding site contains a “C” at position −13 and a “T” at position −14 (Figure 1). Activity of σ^*S*^RNAP promoters is not limited to stationary phase—activity is also induced in response to stresses such as osmotic, heat shock, and low pH (Kazmierczak et al., 2005). Whether, the upstream promoter uses an alternative RNAP or simply responds to environmental stresses remains to be determined.

In our model, aerobic conditions, as well as acidic conditions are stresses that lead to transcription from the upstream putative P1 promoter instead of or in addition to the σ^70^-RNAP, Fe-Fur repressible P2 promoter making iron-regulation negligible under these conditions. However, under microaerobic conditions, transcription from the upstream promoter is repressed. Whether this repression is due to a requirement for a different sigma factor RNAP or a DNA looping mechanism related to the two unidientifed protein binding sites that occludes the upstream promoter is undetermined. However, this leaves the downstream Fur-regulated P2 promoter as the active promoter which can be repressed by iron and Fur. Unlike FeoA, which has high amino acid similarity to FeoA of *E. coli* and *Shigella*, the *feoA* promoter regions of *Y. pestis* and *E. coli* show no significant similarities when compared via a DNA BLAST search (Zhang et al., 2000). Thus, the avoidance of iron-repression aerobically may be unique to *Y. pestis*, at least until the *feoA* promoter regions of other bacteria are assessed under aerobic conditions. Whether this lack of regulation aerobically plays a positive role in *Y. pestis* remains to be determined. One possible role would be that Feo serves to acquire basal levels of iron aerobically even under iron-surplus conditions. *Y. pestis* also appears to be unique in that Fnr, ArcA, RstAB, and FeoC do not regulate the *feoABC* operon (Fetherston et al., 2012) although they are proven regulators of this operon in other enterics (Kammler et al., 1993; Boulette and Payne, 2007; Jeon et al., 2008; Kim et al., 2013).

As expected our transport studies showed that FeoABC and YfeABCD each function to take up iron in an energy-dependent manner under reducing conditions (ferrous) (Figure 7A) Together these two system are responsible for nearly all the iron uptake observed. However, the Fet-Flp system and likely the EfeUOB systems also function in iron uptake. Perhaps neither of these systems functioned significantly during the 30-min time frame or conditions of this experiment. Unexpectedly we found that both the FeoABC and YfeABCD systems could use ferric iron as a substrate for uptake over the 30 min period (Figure 7B). These experiments did not distinguish between the possibility that both systems transport ferric iron or that ferric iron is reduced to ferrous prior to transport. However, either alternative suggests that the FeoABC and YfeABCD transporters could function aerobically under non-reducing conditions.

## Author contributions

LO is the first author. LO and JF contributed experimental work and planning of the research course. RP oversaw the research and contributed to the planning of the research course.

### Conflict of interest statement

The authors declare that the research was conducted in the absence of any commercial or financial relationships that could be construed as a potential conflict of interest.
