# Supplementary material for: The feoABC Locus of Yersinia pestis Likely Has Two Promoters Causing Unique Iron Regulation
Source: Front Cell Infect Microbiol. 2017 Jul 21;7:331. doi: 10.3389/fcimb.2017.00331 (PMC5519574; doi:10.3389/fcimb.2017.00331)
Supplement: Supplementary file 1 [file Table1.pdf]

Supplemental Material

The *feoABC* locus of *Yersinia pestis* likely has two promoters causing unique iron regulation

by

Lauren O'Connor*, Jacqueline D. Fetherston, and Robert D. Perry[a]

Department of Microbiology, Immunology, and Molecular Genetics, University of Kentucky, Lexington, KY, USA

[a] Corresponding author: Robert D. Perry, Department of Microbiology, Immunology, and Molecular Genetics, University of Kentucky, 800 Rose St., Lexington, KY, 40536-0298 USA

Phone: 260-691-0298

e-mail: rperry@uky.edu

* Current address: Department of Human Genetics, University of Michigan Medical School, Ann Arbor, MI, USA

Table S1. Bacterial strains and plasmids used in this study.

Strain or plasmid	Relevant characteristics	Source or reference
Y. pestis		
KIM6+	Pgm$^+$ Yfe$^+$ Feo$^+$ Lcr$^-$	[1]
KIM6	Pgm$^-$ (Δ*pgm*) Yfe$^+$ Feo$^+$ Lcr$^-$	[1]
KIM6-2030	Km^r Pgm$^-$ (Δ*pgm*) Yfe$^+$ Feo$^+$ Fur$^-$ (*fur::kan-9*) Lcr$^-$	[2]
KIM6-2046.1	Km^r Ybt$^-$ (*irp2::kan2046.1*) Lcr$^-$	[3]
KIM6-2088.5	Km^r Ybt$^-$ (*irp2::kan2046.1*) Yfe$^-$ (Δ*yfeAB2031.1*) FeoB$^-$ (Δ*feoB2088*) Lcr$^-$	[4; 5]
KIM6-2088.11	Km^r Ybt$^-$ (*irp2::kan2046.1*) Yfe$^-$ (Δ*yfeAB2031.1*) Feo$^+$ Lcr$^-$	This study
KIM6–2088.17	Km^r Ybt$^-$ (*irp2::kan2046.1*) (Δ*feoB2088*) Lcr$^-$	This study
KIM6-2189 +	Cm^r Pgm$^+$ Yfe$^+$ Feo$^+$ RstAB$^-$ (Δ*rstAB2189::cam*) Lcr$^-$	[4]
KIM6-2203+	Gm^r Pgm$^+$ Yfe$^+$ Feo$^+$ Lcr$^-$ *att*Tn*7:: feoA::lacZ* (integrated 396bp *feoA* reporter); pUCR6KfeoA-lac-Gm integrated into KIM6+	This study
KIM6-2204+	Gm^r Pgm$^+$ Yfe$^+$ Feo$^+$ Lcr$^-$ *att*Tn*7:: feoA165::lacZ* (integrated 165 bp *feoA* reporter); pUCfeoA165-lac-Gm integrated into KIM6+	This study
KIM6-2205+	Gm^r Pgm$^+$ Yfe$^+$ Feo$^+$ Lcr$^-$ *att*Tn*7:: feoA208::lacZ* (integrated 208 bp *feoA* reporter); pUCR6Kfeo208-lac-Gm integrated into KIM6+	This study
KIM6-2206+	Gm^r Pgm$^+$ Yfe$^+$ Feo$^+$ Lcr$^-$ *att*Tn*7:: feoA447::lacZ* (integrated 447 bp *feoA* reporter); pUCR6KfeoP447-lac-Gm integrated into KIM6+	This study
KIM6-2207+	Gm^r Pgm$^+$ Yfe$^+$ Feo$^+$ Lcr$^-$ *att*Tn*7::feoA*Δ*feoP1::lacZ* (integrated *feoA* reporter with P1 promoter region deleted); pUCR6K-ΔfeoP1-lac-Gm integrated into KIM6+	This study
KIM6-2208+	Gm^r Pgm$^+$ Yfe$^+$ Feo$^+$ Lcr$^-$ *att*Tn*7:: feoA*Δ*FBS::lacZ* (integrated *feoA* reporter with P2 promoter region deleted); pUCR6K-ΔfeoFBS-lac-Gm integrated into KIM6+	This study
KIM6-2209+	Gm^r Pgm$^+$ Yfe$^+$ Feo$^+$ Lcr$^-$ *att*Tn*7::feoA*Δ*R1::lacZ* (integrated *feoA* reporter with R1 repeat deleted); pUCR6KfeoΔR1 integrated into KIM6+	This study
KIM6-2210+	Gm^r Pgm$^+$ Yfe$^+$ Feo$^+$ Lcr$^-$ *att*Tn*7::feoA*Δ*R2::lacZ* (integrated *feoA* reporter with R2 repeat deleted); pUCR6KfeoΔR2 integrated into KIM6+	This study
KIM6-2211+	Gm^r Pgm$^+$ Yfe$^+$ Feo$^+$ Lcr$^-$ *att*Tn*7::feoA*Δ*R3::lacZ* (integrated *feoA* reporter with R3 repeat deleted); pUCR6KfeoΔR3 integrated into KIM6+	This study
KIM6-2212+	Gm^r Pgm$^+$ Yfe$^+$ Feo$^+$ Lcr$^-$ *att*Tn*7::lacZ* (integrated *feoA* reporter with R3 repeat deleted); pUCR6K-lac-Gm lacking any promoter region integrated into KIM6+	This study

E. coli		
DH5α (λ pir)	cloning strain	S.C. Straley
**Plasmids**		
pNEBfeoΔR1	deletion of base pairs 356-365 from the *Y. pestis feo* promoter in pNEBfeoP	This study
pNEBfeoΔR2	deletion of base pairs 196-205 from the *Y. pestis feo* promoter in pNEBfeoP	This study
pNEBfeoΔR3	deletion of base pairs 141-150 from the *Y. pestis feo* promoter in pNEBfeoP	This study
pBSfeoPlacZΔRs	High-copy-number plasmid containing the *Y. pestis feo* promoter cloned in front of *lac*Z (Fetherston et al 2012 Infect. Immun.)	[4]
pNEBΔfeoP1	derivative of pNEBfeoP with base pairs 166-185 deleted	This study
pNEBΔfeoFBS	deletion of base pairs 29-69 from the *Y. pestis feo* promoter in pNEBfeoP	This study
pNEBfeoP	*Y. pestis* KIM6+ *feo* promoter region cloned into pNEB193 (Fetherston et al 2012 Infect. Immun.)	[4]
pNEBfeoP447	447 bp fragment from the *feo* promoter region amplified from pWSKfeo and cloned into pNEB193	This study
pUCR6K-ΔfeoFBS-lac-Gm	*feo* promoter from pNEBΔfeoFBS cloned into pUCR6K-lac-Gm	This study
pUCR6K-ΔfeoP1-lac-Gm	*feo* promoter from pNEBΔfeoP1 cloned into pUCR6K-lac-Gm	This study
pUCR6KfeoΔR1	*feo* promoter from pNEBfeoΔR1 cloned into pUCR6K-lac-Gm	This study
pUCR6KfeoΔR 2	*feo* promoter from pNEBfeoΔR2 cloned into pUCR6K-lac-Gm	This study
pUCR6KfeoΔR3	*feo* promoter from pNEBfeoΔR3 cloned into pUCR6K-lac-Gm	This study
pUCR6KfeoA-lac-Gm	The *feo*::*lac*Z fragment from pWSKfeoPlacZΔRs cloned into a *Pme*I/*Xho*I fragment of pUC18R6K-lac-Gm	This study
pUCR6Kfeo208-lac-Gm	*feo*::*lac*Z fragment from pWSKfeoA208::lacZ cloned into a *Pme*I/*Xho*I fragment of pUC18R6K-lac-Gm	This study
pUCR6KfeoP447-lac-Gm	Fragment containing the *feo* promoter from pNEBfeoP447 cloned into pUCR6K-lac-Gm	This study
pUCR6K-lac-Gm	Fragment containing *lac*Z with the MCS from pWSKlacMCS cloned into pUC18R6K-mini-Tn*7*-Gm	This study
pWSKfeo	8.9 Kb *Bam*HI fragment containing the *Y. pestis* KIM6+ *feo* operon cloned into pWSK29	[4]
pWSK*feoA::lacZ*	11.3 Kb; Km^r , *feoA::lacZ*, 412 bp feo promoter fragment in front of *lacZ* cloned into pWSK129	[4]
pWSK*feoA*396*::lacZ*	5'-truncation of the *feo* promoter fragment to 396bp; derived from pWSK*feoA::lacZ*	This study
pWSK*feoA*335*::lacZ*	5'-truncation of the *feo* promoter fragment to 335bp;	This study

	derived from pWSK*feoA::lacZ*	
pWSK*feoA*271*::lacZ*	5'-truncation of the *feo* promoter fragment to 271bp; derived from pWSK*feoA::lacZ*	This study
pWSK*feoA*208*::lacZ*	5'-truncation of the *feo* promoter fragment to 208bp; derived from pWSK*feoA::lacZ*	This study
pWSK*feoA*195*::lacZ*	5'-truncation of the *feo* promoter fragment to 195bp; derived from pWSK*feoA::lacZ*	This study
pWSK*feoA*165*::lacZ*	5'-truncation of the *feo* promoter fragment to 165bp; derived from pWSK*feoA::lacZ*	This study
pWSK*feoA*125*::lacZ*	5'-truncation of the *feo* promoter fragment to 125bp; derived from pWSK*feoA::lacZ*	This study
pWSK$feoA_{E.coli}$::*lac*Z	329 bp PCR fragment of the *E. coli feo* promoter region from DH5α cloned in place of the *Y. pestis feo* promoter in pWSK*feoA*::*lac*Z	This study
pWSKfeoPlacZΔRs	3.8 Kb *feo*A::*lac*Z fragment from pBSfeoPlacZΔRs cloned into pWSK129	This study
pWSKlacMCS	Plasmid in which the *feo* promoter in pWSKfeoPlacZΔRs was replaced by the multi-cloning site from pNEB193	This study
pNEB193	High-copy-number cloning vector	New Enland Biolabs
pTNS2	Helper plasmid carrying the genes necessary for the insertion of constructs into the *att*Tn*7* site; contains R6K origin	[6]
pUC18R6K-mini-Tn*7*-Gm	Vector for integrating sequences into the *att*Tn*7* site; contains an R6K origin of replication	[6]
pWSK129	6.7 Kb; Km^r , low-copy-number cloning vector	[7]

Table S2. Primers used in this study.

Primer	Sequence (5'-3')	Use
attTn7Yp-fwd	TCAGCTGCCACATGTCGAAG	confirm integrated promoters
dfeoFBS-3	GCTTTATTTAATATCTTTTAATTTGCTCGTTTTGGC	construct FBS deletion
dfeoFBS-5	CAAATTAAAAGATATTAAATAAAGCCTTCCAATAGTAACC	construct FBS deletion
dfeoP1-3	TTGCGATCTCGGTGATAAATAGGAACCATACTCATTAG	construct P1 deletion
dfeoP1-5	GTTCCTATTTATCACCGAGATCGCAAATGAGAATGATG	construct P1 deletion
Ecfeo-5'	TATCGTTTAAACTCCACAGCCAAACTCATAA	construct *E. coli feoA* promoter

Ecfeo-3’	TATCGGCGCGCCAGGTGCCTACTTGTTTCTC	construct and confirm *E. coli feoA* promoter
feoΔ20-5	ATTAACTAGAGAAATAATACTGTAATAAG	confirm *feoAΔFBS::lacZ* promoter
FeoΔR3-3	TAAATAGGAACCATTACAGAAATAAGACATTAACTAGAG	construct *feoAΔR3*
FeoΔR3-5	CTTATTTCTGTAATGGTTCCTATTTATCTCTTGTTTTAC	construct *feoAΔR3*
feo208	TATCGTTTAAACTCATTCTCATTTGCG	construct *feoA208*
feo165	TATCGTTTAAACGATAAATAGGAACCA	construct *feoA2165*
Feo125	TATCGTTTAAACATTAACTAGAGAAAT	construct *feoA125*
Feo-Pro1	TATCGTTTAAACAATTTAACAAAATAATTAATCCCTCCTG	construct *feoA447*
Feo-Pro2	TATCGGCGCGCCTCAGCCAAAACGAGCAAATTAAAAG	confirm integration plasmid constructs and integrated promoters; construct truncated promoters; PCR 396 nt probe
FeoP-4	TATCGGCGCGCCATAAGCCTCTTGAAATTTTG	construct and confirm *feoA447* promoter
FeoR1d-3	ATCCCTCCTGATATTTATAAATAATTACGATAGCATTC	construct *feoAΔR1*
FeoR1d-5	GTAATTATTTATAAATATCAGGAGGGATTAATTATTTTG	construct *feoAΔR1*
FeoR2d-3	ATTTAGTACATCACGATCTCGGTAATTTGATGG	construct *feoAΔR2*
FeoR2d-5	ATTACCGAGATCGTGATGTACTAAATCAGTTAATGAATC	construct *feoAΔR2*
integvec-up	AATTCGATCATGCATGAGC	confirm integration plasmid constructs
pfeo1	TATCGTTTAAACCATTCTATACGTTAA	construct *feoA335*
pfeo2	TATCGTTTAAACACTCATCGGTATTTTCAATTTCAG	confirm *feoAΔfeoP1::lacZ*; construct *feoA271*
pfeo3	TATCGTTTAAACCGATCTCGGTAATTTGATGGT	construct *feoA195*
pfeo4F	5Biosg/AATTTAACAAAATAATTAATCCCTCCTG	PCR 396, 251and 126 nt probes
pfeo4R	AACATTATTGAATCACACTGGTTT	PCR 126 nt probe
pfeo-5F	5Biosg/ACTCATCGGTATTTTCAATTTCAG	PCR 125 nt probe
pfeo-5R	TCTTGTTTTACCATCAAATTACC	PCR 251 and 125 nt probes
pfeo-F6	5Biosg/GATAAATAGGAACCATACTCATTAGTTACAGAAAT	PCR 145 nt probe

pfeo-R6	TCAATTAAATAAAGCCTTCCAATAGTAACC	confirm *feoA*Δ*feoP1::lacZ* construct; PCR 145 nt probe
reverse universal	AGCGGATAACAATTTCA	PCR multiple cloning site (MCS)
universal -40	GTTTTCCCAGTCACGAC	PCR MCS; confirm promoter and plasmid constructs

Table S3. Three 11 bp direct repeat sequences (R1-R3) in the *feoA* promoter region.

R1	ATTCTCATTAG
R2	ATTCTCATTTG
R3	ATACTCATTAG
Consensus	AT(t/a)CTCATT(a/t)G

Fig. S1. The *E. coli feoA* promoter is iron repressed in *Y. pestis.* KIM6+ carrying the *feo*$_{E. coli}$*::lacZ* reporter plasmid was grown in the presence or absence of 10μM $FeCl_3$ at 37°C under aerobic conditions in cPMH2. Samples were taken during mid-exponential phase for β-galactosidase assays. Activities are averages of multiple samples from at least two independent experiments expressed in Miller units. Error bars represent standard deviations. The statistically significant fold difference due to iron status is shown (P = <0.0002 [***]).

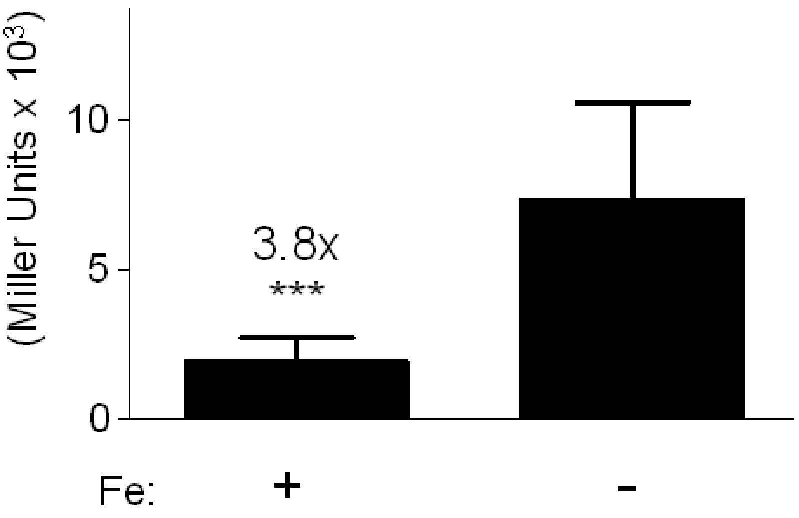

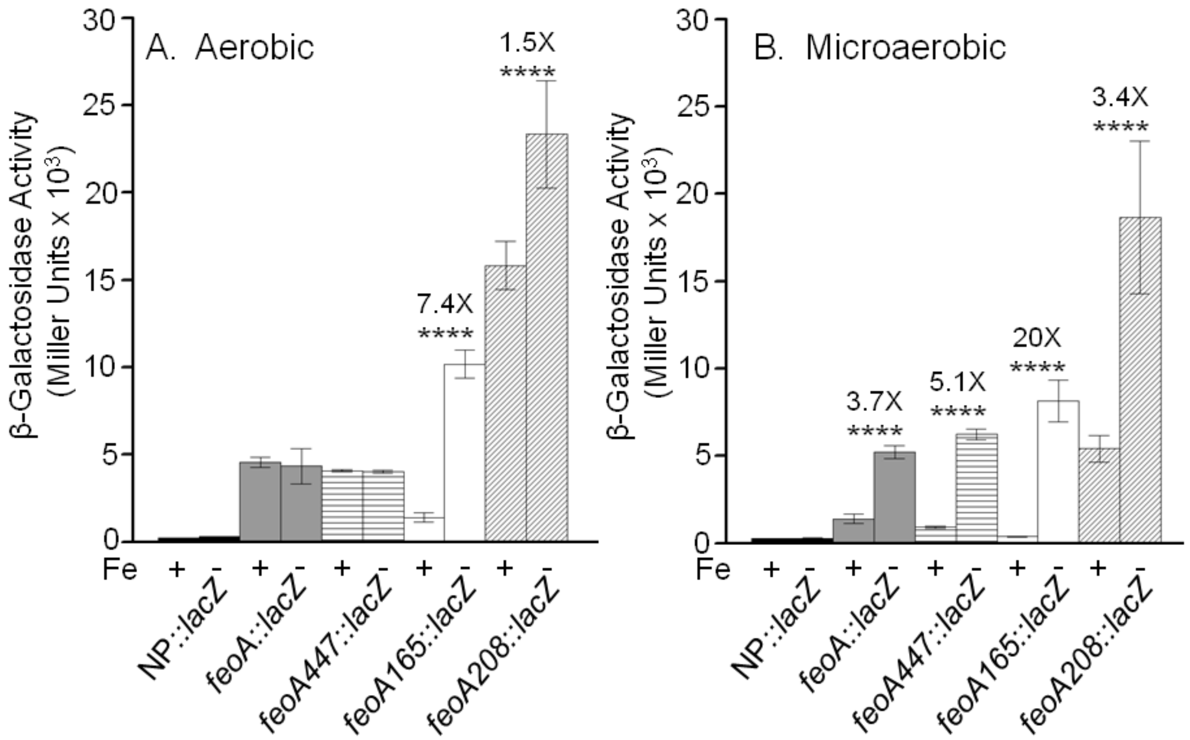

Fig. S2. Transcriptional expression patterns from full-length and truncated *feoA* reporters are not due to titration of transcriptional regulators. Transcription from the *att*Tn*7*::*feoA*::*lacZ* reporter (396 bp) (KIM6-2202+) is compared to transcription from the *att*Tn*7*:: *feoA165*::*lacZ* (KIM6-2204+), *att*Tn*7*:: *feoA208*::*lacZ* (KIM6-2205+), and *att*Tn*7*:: *feoA447*::*lacZ* (KIM6-2206+) reporters. KIM6-2212+ (*att*Tn*7*::*lacZ* ; no promoter region – NP::*lacZ*) is used as a negative control. All strains were grown in the presence or absence of 10µM $FeCl_3$ at 37°C under aerobic or microaerobic conditions in cPMH2. Samples were taken during mid-exponential phase for β-galactosidase assays. Activities are averages of multiple samples from at least two independent experiments expressed in Miller units. Error bars represent standard deviation. Values for NP::*lacZ* and *feoA*::*lacZ* are from Fig. 6. Regulatory patterns of all integrated constructs were the same as their plasmid counterparts (Fig. 2).

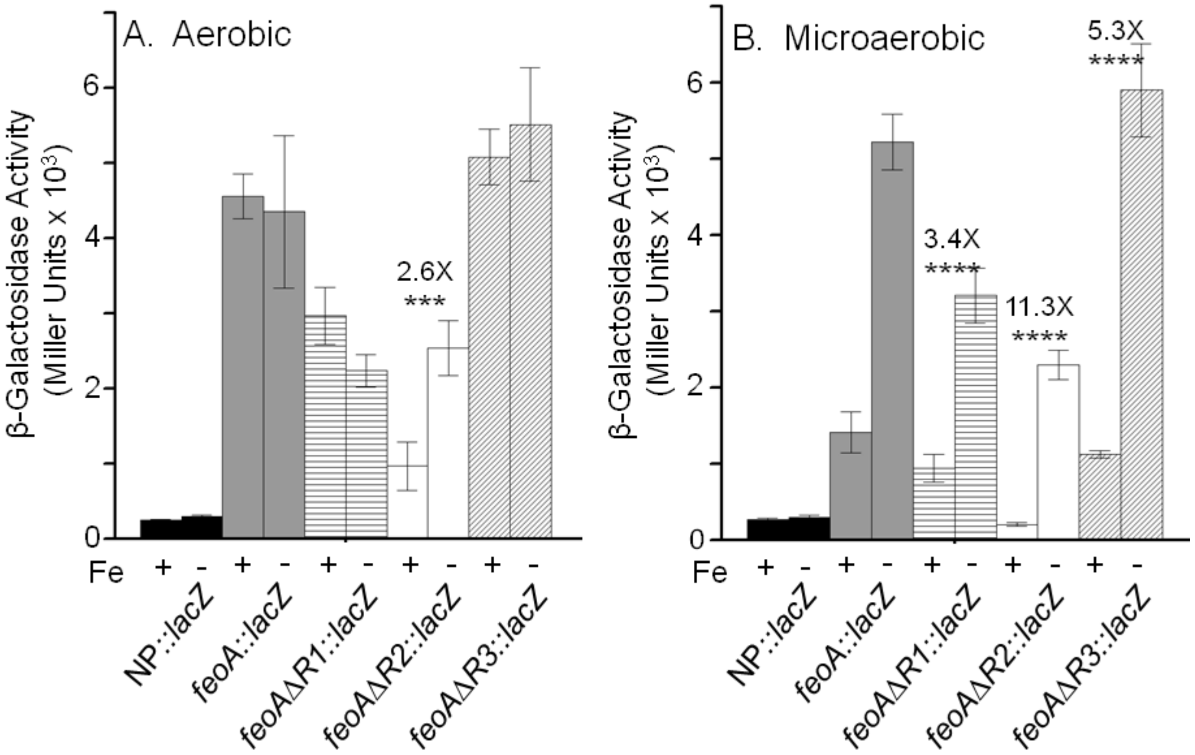

Fig. S3 Transcriptional activities of integrated *feoA* promoters with deletions of direct repeats R1, R2, or R3. Transcription from the *att*Tn*7*::*feoA*::*lacZ* reporter (KIM6-2202+) (values from Fig. 6) is compared to transcription from the *attTn7::feoA*Δ*R1::lacZ* (KIM6-2209+) and *attTn7::feoA*Δ*R2::lacZ* (KIM6-2210+) reporters and *attTn7::feoA*Δ*R3::lacZ* (KIM6-2211+) reporters. Negative control KIM6-2212+ (*att*Tn*7::lacZ* ; no promoter region) (values from Fig. 6) is used as a negative control. All strains were grown in the presence or absence of 10μM $FeCl_3$ at 37°C under aerobic or microaerobic conditions in cPMH2. Samples were taken during mid-exponential phase for β-galactosidase assays. Activities are averages of multiple samples from at least two independent experiments expressed in Miller units. Error bars represent standard deviation. The statistically significant fold difference due to iron status is shown (P = 0.0003 [***]; $P \leq$ 0.000005 [****].

REFERENCES

[1] Sikkema, D.J., and Brubaker, R.R. (1987). Resistance to pesticin, storage of iron, and invasion of HeLa cells by yersiniae. *Infect. Immun.* 55, 572-578.

[2] Staggs, T.M., and Perry, R. D. (1991). Identification and cloning of a *fur* regulatory gene in *Yersinia pestis*. *J. Bacteriol.* 173, 417-425.

[3] Fetherston, J.D., Lillard, J.W., Jr., and Perry, R.D. (1995). Analysis of the pesticin receptor from *Yersinia pestis*: role in iron-deficient growth and possible regulation by its siderophore. *J. Bacteriol.* 177, 1824-1833.

[4] Fetherston, J.D., Mier, I., Jr., Truszczynska, H., and Perry, R.D. (2012). The Yfe and Feo transporters are involved in microaerobic growth and the virulence of *Yersina pestis* in bubonic plague. *Infect. Immun.* 80, 3880-3891.

[5] Perry, R.D., Mier, I., Jr., and Fetherston, J.D. (2007). Roles of the Yfe and Feo transporters of *Yersinia pestis* in iron uptake and intracellular growth. *BioMetals* 20, 699-703.

[6] Choi, K.-H. , Gaynor, J.B., White, K.G.,. Lopez, C , Bosio, C.M., Karkhoff-Schweizer, et al. (2005). A Tn*7*-based broad-range bacterial cloning and expression system. *Nat. Methods* 2, 443-448.

[7] Wang, R.F., and Kushner, S.R. (1991). Construction of versatile low-copy-number vectors for cloning, sequencing and gene expression in *Escherichia coli*. Gene 100, 195-199.
